# Comparative evaluation of two MALDI-TOF MS systems for microbial identification: accuracy and workflow efficiency in a clinical microbiology laboratory

**DOI:** 10.1128/spectrum.02981-25

**Published:** 2026-01-30

**Authors:** Jasmin Köffer, Jale Ören, Ulrike Betz, Janhendrik Timm, Meike Oftring, Nina Bauer, Maxime Ndolumingo, Lelia Abad, Ulrich Eigner

**Affiliations:** 1 MVZ Laboratory Dr. Limbach 39289 , Heidelberg, Germany; 2 bioMérieux 1896 , Marcy-l'Étoile, France; Boston Medical Center, Boston, Massachusetts, USA

**Keywords:** MALDI-TOF MS, rapid identification, MALDI Biotyper Sirius, VITEK MS PRIME, workflow

## Abstract

This study aimed to compare the identification accuracy and workflow of the VITEK MS PRIME (VMP) and the MALDI Biotyper Sirius (BS) in a routine laboratory setting. A total of 927 isolates, representing 219 different species, were analyzed using both the VMP and the BS system. In addition, 119 positive blood cultures were tested after 4–6 h of subculture, with and without formic acid, and compared to the results obtained after 18 to 24 h. Hands-on time (HOT) and time to result (TTR) were measured based on a workflow consisting of 16 slides, each containing 16 isolates, with a total of 256 isolates, simulating the daily workload of a single technician. Of the 927 isolates tested, both systems demonstrated high identification rates (91.1% for BS and 95.5% for VMP). Agreement between the two matrix-assisted laser desorption/ionization time-of-flight (MALDI-TOF) instruments after retesting was 99.4% and 97.5% at genus and species levels. In short-incubation cultures, VMP identified 118 (99.2%) isolates without and 113 (95.0%) with formic acid treatment, while BS identified 105 (88.2%) and 110 (92.4%), respectively. Workflow analysis demonstrated a HOT of 106 min and a TTR of 191 min for VMP with 252 successful identifications (98%). In contrast, BS required 155 min for both HOT and TTR, yielding 211 successful identifications (82%). Both MALDI-TOF systems evaluated in this study demonstrated an excellent concordance in the identification of a broad range of species. Depending on the laboratory settings, either system is well-suited to support an efficient and streamlined laboratory workflow.

**IMPORTANCE:**

This study provides a real-world comparison of two leading matrix-assisted laser desorption/ionization time-of-flight mass spectrometry (MALDI-TOF MS) systems, VITEK MS PRIME (VMP) and MALDI Biotyper Sirius (BS), based on 927 isolates across 219 species, including rare and challenging organisms. By simulating routine lab workflows working with several workstations, it offers practical insights into hands-on time and time to results. Unlike previous studies, it includes a broader sample set (including yeasts), evaluates short positive blood culture incubation conditions, and accounts for operator variability. These strengths make it a valuable resource for laboratories seeking evidence-based guidance on MALDI-TOF implementation and optimization.

## INTRODUCTION

Matrix-assisted laser desorption/ionization time-of-flight mass spectrometry (MALDI-TOF MS) enables the identification of bacteria and fungi from microbial cultures based on their protein spectra. This technology has largely replaced traditional biochemical methods and is now considered the gold standard for the identification of bacterial and yeast isolates (1). MALDI-TOF MS is a rapid and cost-effective method and offers the possibility of accurate identification of a wide range of clinically relevant microorganisms (2, 3). Fast and reliable pathogen identification using MALDI-TOF significantly contributes to targeted therapy and cost savings for health care systems (4–7). In clinical laboratories, MALDI-TOF is also used for the identification of bacterial isolates from positive blood cultures. To further shorten turnaround times, various protocols have been developed for pathogen identification directly from positive blood culture samples or from microcolonies obtained after short incubation periods (2–6 h) (8). In times of staff shortages, limited budgets, and increasing sample numbers, laboratories are increasingly required to optimize workflows and implement automation strategies (9). MALDI-TOF plays a crucial role in these improvements, particularly in medium to large-sized microbiology laboratories. There are several MALDI-TOF platforms used in microbiological laboratories worldwide. Two of the most important MALDI-TOF systems are the new VITEK MS PRIME (bioMérieux) and the MALDI Biotyper Sirius (Bruker Daltonics). Several publications have demonstrated the high accuracy of both systems for identifying a broad spectrum of microorganisms, including anaerobes, mycobacteria, yeasts, and filamentous fungi (10–15). A recent time-motion study by Thelen et al. compared both instruments under different workflow scenarios, highlighting differences in performance depending on laboratory size and operational requirements (9).

To understand the added value of using these instruments in an urban clinical laboratory with medium to high-throughput of identifications, we conducted a study comparing the performance, workflow, and usability of these instruments, based on local epidemiology, rapid identification protocols, and laboratory organization.

## MATERIALS AND METHODS

### Study design

This study was conducted at MVZ Labor Dr. Limbach & Kollegen eGbR in Heidelberg, Germany. The objective was to compare the performance and the workflow efficiency of the VITEK MS PRIME (VMP) and the MALDI Biotyper Sirius (BS) systems. The study was therefore divided into three independent experiments. In the first part, the performance of the systems was evaluated using prospective and retrospective samples from agar culture. For sample selection, prospective isolates from routine operations were initially collected, with no more than approximately 10 samples per species to ensure coverage of a broad diversity. To guarantee inclusion of less common pathogens, this initial set was further supplemented with retrospective isolates. The agreement of the results was assessed at both the species and genus levels in cases where both systems provided a paired identification result in parallel. For samples with discordant results, the respective isolates were sent for sequencing. In the second part, the identification accuracy for microcolonies from positive blood cultures was compared after a short culture (4–6 h) and after a long culture (18–24 h). The final part involved a workflow analysis comparing the hands-on time (HOT) and time to result (TTR) of the two devices.

To show that the performance of the systems is not influenced by the operator’s experience, the first two study parts were performed with two operators, one with less than 1 year of MALDI-TOF MS experience and another with over 5 years’ experience with MALDI-TOF MS, respectively. To ensure comparability, the same operator performed the tests for each sample on both systems.

### Culture conditions

Prospective samples were randomly collected from various sample types (urine, respiratory, skin, etc.). The retrospective isolates were revived from −80°C storage. All isolates were cultured on appropriate media at 35°C to 37°C under suitable conditions (18–24 h for aerobic bacteria and 24–48 h for anaerobic bacteria). The culture media used were Columbia agar with 7% sheep blood (Oxoid, Thermo Fisher Scientific, Waltham, MA, USA), chocolate agar supplemented with PolyViteX (bioMérieux, Marcy l’Étoile, France), and MacConkey II agar (Becton Dickinson, Sparks, MD, USA). Positive blood culture specimens were incubated in the BD BACTEC FX automated system (BD BACTEC; BD Biosciences, Sparks, MD, USA) at 35°C. To create a subculture, bacteria-positive blood cultures were subcultured with one drop of blood on Columbia blood agar (ThermoFisher Scientific, USA) and incubated under suitable conditions. Microcolonies were analyzed after 4–6 h and 18–24 h of incubation on both systems.

### Sample preparation

**Fig 1:**
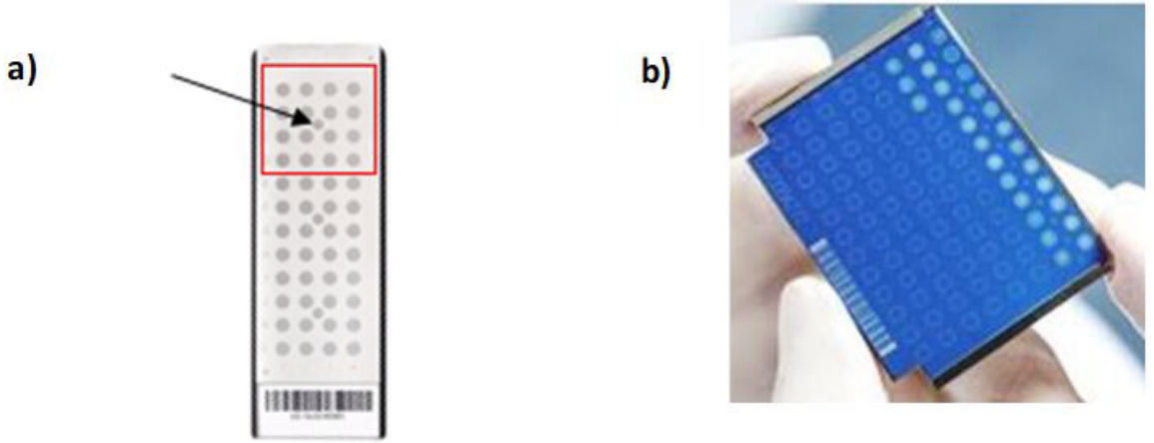
Disposable MALDI target plates (**a**) for VITEK MS PRIME, with acquisition group (red) and calibrator spot (arrow) (**b**) for Biotyper Sirius in an AnchorChip.

For VMP, the VITEK PICKME pen was used to prepare the target slides. A volume of 1.0 µL of a saturated solution of alpha-cyano-4-hydroxycinnamic acid matrix in 50% acetonitrile and 2.5% trifluoroacetic acid (VITEK MS-CHCA, bioMérieux) was applied to the spot with a pipette. For yeast isolates, 0.5 µL of 25% formic acid (VITEK MS-FA, bioMérieux) was added. Instrument calibration was performed daily following the standard procedure for bacteria. *Escherichia coli* ATCC 8739 calibrator strain was applied onto the VITEK MS-DS in the three designated positions. As a calibrator, the *E. coli* ATCC 8739 strain calibrator was spotted with each acquisition group on VITEK MS-DS (Fig. 1a). Additional positive and negative controls were included on each day of testing. The quality control strain *Klebsiella aerogenes* ATCC 13,048 was used as a positive control, and 1.0 µL of matrix reagent was used as a negative control. For BS, colonies were applied on a spot of the BS biotarget plate (MBT Biotarget 96, Bruker Daltonics, Bremen, Germany) using a single-use loop by direct smear. A 1.0 µL volume of IVD HCCA-matrix (Bruker) was added to each spot. For yeast identification, 1 µL of 70% formic acid was applied. For each target on the BS, the IVD Bacterial Test Standard (BTS) (Bruker) was used as a calibrator.

For the second part of the study, microcolonies from positive blood cultures were identified using both systems, with and without the application of formic acid.

### Result interpretation and discordant results

Each isolate was identified in parallel using VMP (Software Version 1.1.2; IVD Knowledge Base V3.2) and the BS system (Software Version 5.2.300; Server Version 4.1.100, MBT Compass Library 2023). The identification rate of the systems was determined under the following criteria: with the VMP, a confidence value of 99.9% was considered as high-confidence identification; 60% to 99.8% as low discrimination, and <60% as no identification. For BS, a log score of ≥2.0 was considered a high-confidence identification; a log score between 1.7 to 1.99 as low-confidence; and a log score <1.7 as no identification. Testing of unidentified isolates was repeated once with the respective IVD software. If the isolate was still not identified after re-analysis, the isolate was tested once in the RUO database. Polymicrobial cultures were excluded from analysis.

### Sequencing

For the resolution of discrepant identification results, next-generation sequencing (NGS) using the Illumina Novaseq 9000 (Illumina) PE150 platform and Sanger Sequencing (16S-rDNA-, *tuf-*, *rpoB*-, *gyrB*-genes) on the Life Technology AB3730XL instrument was performed by BaseClear B.V. (the Netherlands). The respective isolates were cultivated on blood agar plates and inoculated in Shield solution (Zymo Research) before sequencing. The FastANI algorithm (16) (version 1.33) was used to calculate the average nucleotide identity between the Illumina *de novo* assembled query genome (the strain of interest) and a database of 53,836 reference genomes. The reference genomes were retrieved from the NCBI Genome database using the following selection criteria: “bacteria,” assembly level “complete” or “chromosome,” exclude “atypical.”

### Workflow analysis

For workflow comparison of the two MALDI-TOF systems, HOT, including target preparation, drying time, and target loading, was monitored. Additionally, TTR, including system measurement, was evaluated. The workflow comparison was conducted over one working day with two operators (one for the VMP and one for the BS). The analysis simulated the routine workflow of Laboratory Limbach. A total of 16 target slides with 16 spots each were prepared and measured sequentially (one by one) on both systems. In total, 256 isolates were measured per system, reflecting the number of daily identifications per technician in our lab. The detailed workflow steps considered for comparison are outlined in Table 1.

**TABLE 1:** Steps considered for workflow comparison of VITEK MS PRIME and Bruker MALDI Biotyper Sirius

	VITEK MS PRIME	Bruker MALDI Biotyper Sirius
Slide preparation	Scanning the slide into VITEK FLEXPREP system and name the spots.	Drop the BTS on the spot, using a pipette
	Prepare the ATTC spot, using VITEK MS PICKME. Collecting the colonies from the plate, using VITEK MS PICKME and smear the spots and drop matrix after each spot	Collecting the colonies from the plate, using a disposable loop and smear the spots. The Matrix will be spotted for all spots at once within the period of 30 min
Dry time	Drying time need for matrix before loading	Drying time need for matrix before loading
	During drying time, the smearing of the next slide can be started	During drying time, the smearing of the next slide can be started
Loading	Load the slide into the LOAD&GO carrier	Add slide information to the Bruker Software Version 5.2.300. Open the system, load the slide, close the system, and wait for the BTS spot to be validated
System run	Time start measurement until finish group	Time start measurement until finish group
	Time measured and evaluated by the system in the VITEK MS Software	No access to the system times therefore measurement will be done manually
Issues	If a measurement of the ATTC strain fails, the whole run fails and will be recorded as an unsuccessful measurement	If a measurement of the BTS strain fails, a new BTS spot can be dripped, and the slide can be reloaded into the system. Reloading time was added to the total loading time. If the BTS measurement fails again the run and will be recorded as an unsuccessful measurement

### Statistical methods

Differences in identification rate were analyzed by the chi-square test. Findings were considered significant if the *P* value was <0.05. Statistical analysis was performed using GraphPad Prism version 10.0, GraphPad Software, Inc., San Diego, CA.

## RESULTS

### Performance comparison using clinical isolates

A total of 927 isolates (511 retrospectives and 416 prospective samples) representing 219 different species were analyzed. No difference in terms of performance was observed between operators, regardless of their level of experience. Identification results were obtained for 885 isolates (95.5%) using the VMP and for 844 (91.1%) isolates using the BS (*P* < 0.001) (Table 2). The VMP failed to identify 27 isolates, while the BS failed to identify 68 isolates; 15 isolates were not identified by both systems. Finally, identification results were available for 817 isolates from both devices in parallel, allowing for a direct comparison of identification agreement (Table 3). Among the identified species, 437 (53%) were Gram-positive bacteria, 350 (43%) were Gram-negative bacteria, and 30 (4%) were yeasts. Agreement between the systems at the genus and species levels was 99.4% and 97.5%, respectively. Discordant identification results were observed in 5 of 817 (0.6%) isolates at the genus level, and 15 of 817 (1.8%) isolates at the species level. Sequencing was performed on the 20 samples with discrepant identification results, revealing misidentification in 10 (1.2%) isolates using VMP and 11 isolates (1.3%) using BS. Sequencing was not able to differentiate species between *Pantoea agglomerans* and *Pantoea septica*. The performance of the VMP and BS systems across different bacterial groups and yeasts is presented in Tables 2 and 3, and details of discordant results are summarized in Table 4. Throughout the study, no differences were observed between experienced and inexperienced operators in terms of accuracy.

**TABLE 2:** Identification rate and agreement of the VMP and BS for the detection of pathogens inoculated on agar plates

	VMP, *n* (%)	BS, *n* (%)	*P* value
Total no. of isolates	927		
Isolates with ID available	885 (95.5%)	844 (91.1%)	<0.0001
Isolates with no ID in	27	68	
Isolates with no ID in both instruments	15	15	
Score >2.0		658 (78.0%)	<0.0001
Score 1.7–1.99		186 (22.0%)	
Conf. value 99.9%	817 (92.3%)		
Conf. value 60%–99.8%	68 (7.7%)		
Isolate ID-pairs compared	817		
Agreement of genus level	812 (99.4%)		
Agreement of species level	797 (97.5%)		

**TABLE 3:** Performance results of the VMP and BS system by bacterial groups and yeasts^*a*^

Groups	Isolates, *n*	VMP, *n* (%)		BS, *n* (%)		*P* value for correct ID
		Correct ID *n* (%)	Mis ID *n* (%)	Correct ID *n* (%)	Mis ID *n* (%)	
gram+ cocci	328	327 (99.7)	1 (0.3)	326 (99.4)	2 (0.6)	0.6
gram+ rods	109	105 (96.3)	4 (3.7)	105 (96.3)	4 (3.7)	0.7
gram− rods	337	332 (98.5)	5 (1.5)	332 (98.5)	5 (1.5)	1
gram− cocci	13	13 (100)	0 (0)	13 (100)	0 (0)	1
Yeast	30	30 (100)	0 (0)	30 (100)	0 (0)	1
Total	817	807 (98.8)	10 (1.2)	805 (98.5)	11 (1.5)	0.6

^*a*^ Specimens for which one or both systems showed no ID result were not included in the table.

**TABLE 4:** Details of discordant results

Vitek MS prime	Value	Biotyper Sirius	Value	Sequencing	%
*Staphylococcus haemolyticus*	99.9	*Staphylococcus borealis*	2.29	*Staphylococcus haemolyticus*	99.48
*Staphylococcus haemolyticus*	79.0	*Staphylococcus petrasii*	2.23	*S. jettensis/S. petrasii*	99.46
*Staphylococcus haemolyticus*	99.9	*Staphylococcus borealis*	2.48	*Staphylococcus haemolyticus*	98.81
*Dermabacter hominis*	99.9	*Corynebacterium amycolatum*	1.82	*Corynebacterium amycolatum*	96.96
*Dermabacter hominis*	99.9	*Corynebacterium amycolatum*	2.14	*Corynebacterium amycolatum*	98.44
*Listeria monocytogenes*	99.9	*Listeria welshimeri*	1.82	*Listeria monocytogenes*	99.47
*Listeria monocytogenes*	99.9	*Listeria innocua*	1.84	*Listeria monocytogenes*	100
*Listeria monocytogenes*	97.7	*Listeria innocua*	1.94	*Listeria monocytogenes*	100
*Listeria monocytogenes*	99.9	*Listeria innocua*	2.18	*Listeria monocytogenes*	99.65
*Weissella confusa*	99.9	*Weissella cibaria*	2.29	*Weissella cibaria*	98.69
*Bacillus cereus group (RUO)*	99.9	*Bacillus thuringiensis*	1.82	*Ralstonia* sp.	98.19
*Parabacteroides distasonis*	99.9	*Bacteroides cellulosilyticus*	2.09	*Parabacteroides distasonis*	97.41
*Enterobacter* sp. *(RUO)*	89.9	*Raultella ornithinolytica/Raultella planticola*	1.97	*Raoultella planticola*	99.91
*Escherichia coli*	97.7	*Escherichia marmotae*	2.13	*Escherichia marmotae*	99.53
*Citrobacter freundii*	99.9	*Citrobacter braakii*	1.74	*Citrobacter freundii*	99.82
*Klebsiella pneumoniae*	99.9	*Klebsiella variicola*	2.26	*Klebsiella variicola*	99.90
*Klebsiella pneumoniae*	99.9	*Klebsiella variicola*	1.98	*Klebsiella variicola*	99.70
*Xenorhabdus* sp. *(RUO)*	75	*Kluyvera cryocrescens*	1.77	*Pantoea* sp.	97.04
*Acinetobacter pittii*	99.9	*Acinetobacter lactucae/Acinetobacter nosocomialis*	2.03	*Acinetobacter pittii*	99.73
*Pantoea agglomerans*	99.6	*Pantoea septica*	2.39	*Pantoea* sp.	99.2

### Performance on short and standard incubation using blood cultures

After 4–6 h of incubation, the VMP identified 118 (99.2%) isolates without formic acid and 113 (95.0%) with formic acid, while the BS identified a significantly lower number, 105 (88.2%) isolates without and 110 (92.4%) with formic acid. After 18–24 h, 119 (100%) isolates showed a correct identification result with the VMP and 118 (99.2%) with the BS. The data in the above paragraph is shown in Table 5. The results indicate that the VMP achieved significantly better outcomes with short cultures (*P* < 0.001). The BS primarily faced challenges in identifying Gram-positive bacteria, particularly *Staphylococcus* species (Table 6).

**TABLE 5:** Identification results of the BS and VMP systems for blood cultures inoculated on solid media after different incubation times

Parameters	VMP, *n* (%)	BS, *n* (%)	*P* value
Specimens in total	119		
Detected in short culture (4–6 h)	118 (99.2)	105 (88.2)	<0.001
Detected in short culture (4–6 h) with formic acid	113 (95.0)	110 (92.4)	0.4
Detected in standard culture (18–24 h)	119 (100)	118 (99.2)	1

**TABLE 6:** Species recovered from positive blood culture after short incubation time with and without formic acid and long incubation time^*a*^

Species	*n*	VMP			BS		
		SC with fa	SC without fa	LC	SC with fa	SC without fa	LC
*Acinetobacter pittii*	1	1	1	1	1	1	1
*Acinetobacter ursingii*	1	1	1	1	0	0	1
*Bacillus cereus group*	1	1	1	1	1	1	1
*Citrobacter freundii*	2	2	2	2	2	2	2
*Citrobacter koseri*	2	2	2	2	2	2	2
*Citrobacter youngae*	1	1	1	1	1	1	1
*Corynebacterium striatum*	1	1	1	1	0	0	1
*Enterobacter hormaechei*	5	**3**	5	5	5	5	5
*Enterococcus faecalis*	8	8	8	8	8	6	8
*Enterococcus faecium*	3	3	3	3	3	3	3
*Escherichia coli*	20	20	20	20	20	20	20
*Klebsiella oxytoca*	3	3	3	3	3	3	3
*Klebsiella pneumoniae*	9	**8**	9	9	9	9	9
*Proteus mirabilis*	6	6	6	6	6	6	6
*Serratia marcescens*	1	1	1	1	1	1	1
*Staphylococcus aureus*	11	11	11	11	11	10	11
*Staphylococcus capitis*	4	4	4	4	3	2	4
*Staphylococcus epidermidis*	20	20	20	20	20	17	20
*Staphylococcus haemolyticus*	1	1	1	1	0	1	1
*Staphylococcus hominis*	2	2	2	2	2	2	2
*Staphylococcus lugdunensis*	1	1	1	1	1	0	1
*Staphylococcus pettenkoferi*	1	0	1	1	0	0	1
*Staphylococcus warneri*	3	3	3	3	3	3	3
*Streptococcus dysgalactiae*	1	1	1	1	1	1	1
*Streptococcus gordonii*	7	5	6	7	3	5	6
*Streptococcus mitis/* *Streptococcus oralis*	2	2	2	2	2	2	2
*Streptococcus parasanguinis*	2	2	2	2	2	2	2

^*a*^ SC, short culture (4–6 h); LC, long culture (18–24 h); fa, formic acid.

### Workflow analysis

**Fig 2:**
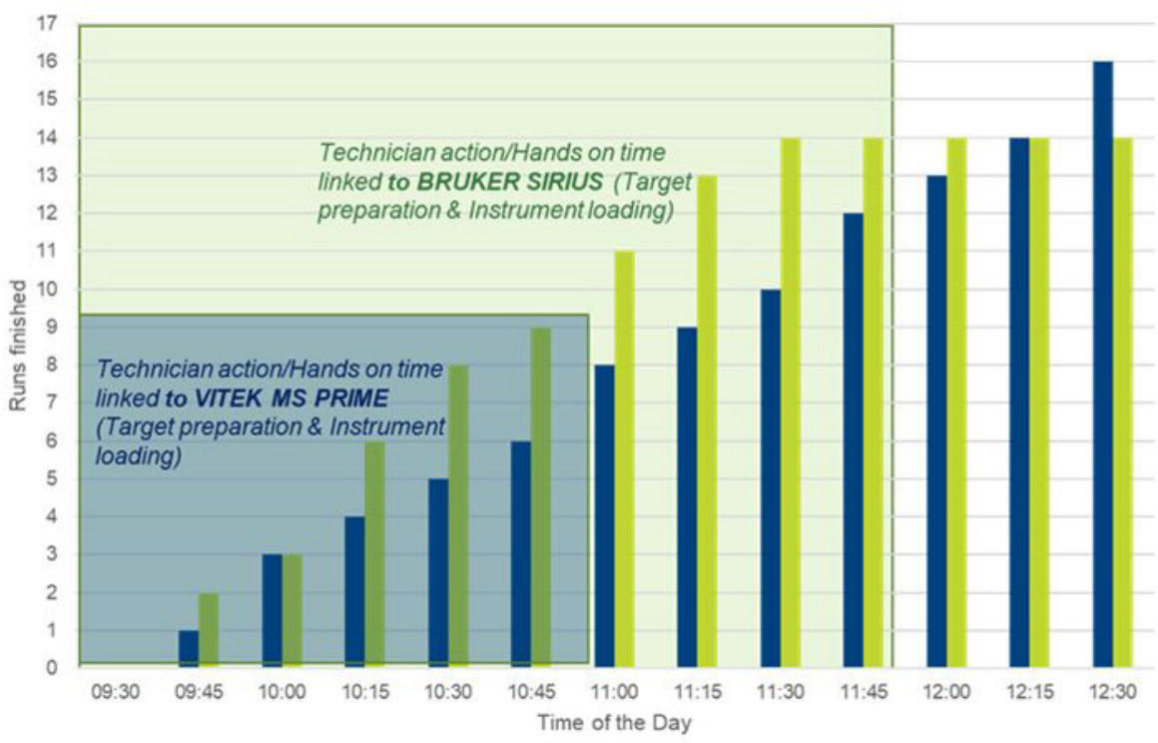
Workflow comparison of the VITEK MS PRIME and Bruker MALDI Biotyper Sirius systems during the day times (HOT and TTR). Blue column: number of finished runs with VMP; green column: number of finished runs with BS; blue square: technician HOT required to launch the runs with VMP; green square: technician HOT required to launch the runs with BS.

The TTR from process initiation to final ID was 36 min longer for the VMP than for the BS. The HOT bounded to the process was 49 min shorter with the VMP. While TTR per identified isolates was comparable, the HOT per identified isolates was halved for the VMP when compared to the one observed with BS. The VMP achieved a higher identification rate compared to BS (98% vs 82%; *P* < 0.0001) (Tables 7 and 8). In Fig. 2, the completed runs are plotted against the time axis. Each run was performed on a slide containing 16 individual sample spots. The blue bars indicate the completed runs for the VMP, while the green bars represent those for the BS. The shaded squares highlight the HOT for each respective device. The bars show that BS processes each target more rapidly; however, the overall HOT for the BS system exceeds that of the VMP, as illustrated by the shaded squares. On the VMP, all 16 slides, which can be accommodated simultaneously, were loaded within 1.5 h and then automatically processed by the system. The manual process took place between 09:30 h to 11:00 h. In contrast, the BS allows one target plate to be loaded at a time, resulting in a longer manual process, which lasted from 09:30 to 11:45. Two targets could not be analyzed using the BS due to repeated BTS acquisition failures, whereas all slides were successfully acquired with the VMP.

**TABLE 7:** Workflow assessment overview^*a*^

	VMP	BS
Isolates identified	252/256 (98%)	211/256 (82%)
Total time to result (min)	191	155
Hand on time (bounded to process) (min)	106	155
HOT/identified isolates (s)	25	44
Total time/identified isolate (s)	45	44

^*a*^ HOT, hands-on time.

**TABLE 8:** Workflow comparison—time of different process steps in minutes—for one slide of 16 spots^*a*^^,^^*b*^

Average time to prepare one target (16 spots) (min)	Total time		HOT		System time	
	VMP	BS	VMP	BS	VMP	BS
Slide prep	5.5	6.8	5.5	6.8	–	–
Dry time	6.1	8.5	6.1	8.5	–	–
Loading	5.2	2	–	2	5.2	–
System run	5.3	2.5	–	–	5.3	2.5
Total	22.1	19.8	11.6	17.3	10.5	2.5

^*a*^ TTR, time to result; HoT, hands-on time.

^*b*^ Wells with “–“ are not applicable.

## DISCUSSION

This study evaluated and compared the performance of the MALDI TOF systems, VITEK MS PRIME (bioMérieux), and the MALDI Biotyper Sirius (Bruker Daltonics) with respect to identification rates and workflow efficiency in routine laboratory settings.

Both systems demonstrated high identification accuracy across a broad spectrum of 219 different bacterial and fungal species, achieving identification rates of 95.5% for VMP and 91.1% for BS. These findings are consistent with Thelen et al., the only publication to date comparing Sirius and VITEK MS PRIME, the two latest versions of MALDI-TOF systems from Bruker and bioMérieux (9). Identification rates observed were 95% and 93.7% for the BS and VMP, respectively (9). In our study, the VMP system demonstrated a high confidence level (>99%) in 92.3% of the samples, while the BS system achieved a score value ≥2.0 in 78% of the samples. These results are similar for VITEK MS PRIME (91.9%), but lower for BS (95%), compared to the results from Thelen et al. (9). This could be explained by the protocol followed in our study: in cases of No ID, each isolate was retested, and if the No ID persisted, the RUO database was used. This approach improved the identification rate for VMP, but not significantly for Sirius. Isolates recovered by VITEK MS PRIME but not by Sirius are shown in Table S1. Additionally, in 8.9% and 4.5% of samples analyzed with BS and VMP, no organism could be identified, corresponding to higher levels than those previously published (1.0% and 1.6%) (9). The lower number of unidentified cases reported is likely due to the difference in species distribution (majority of Gram-negative rods) and the use of routine isolates, rather than the diagnostically challenging specimens included in the present study (9).

Among the 817 samples for which both systems showed a paired identification result, 15 (1.8%) demonstrated discordant results at the species level, and 5 (0.6%) at the genus level. After sequencing resolution, VMP gave good identification in 10 cases of 20 discordances and BS in 9 cases, and 2 were misidentifications at the genus level for both instruments (*Pantoae* sp. detected as *Xenorhabdus* by VMP and *Kluyvera cryocrescens* by BS; *Ralstonia* sp. detected as a *Bacillus* sp. by VMP and BS). The most frequent discrepancies in species identification were observed among several Staphylococcus species (*S. borealis, S. haemolyticus,* and *S. petrasii*), as well as between *Listeria monocytogenes*/*L. innocua* and *Klebsiella pneumoniae*/*variicola*. These species are closely related and exhibit highly similar mass spectral profiles, which likely contribute to the observed identification discrepancies. The differentiation of *Staphylococcus* species, particularly *S. borealis, S. haemolyticus,* and *S. petrasii*, remains challenging. Even with sequencing, distinguishing *S. haemolyticus* from *S. borealis* is difficult, as all housekeeping genes, except the 16S rRNA gene, are identical (17). Similarly, *L. innocua* is closely related to *L. monocytogenes*, but unlike *L. monocytogenes*, it is nonpathogenic to mammals (18), making this distinction clinically significant for accurate diagnosis and patient management. Furthermore, the pathogenicity of *S. borealis* and *S. petrasii* remains poorly understood, as these species were only recently described (19, 20). Likewise, *K. variicola* identification is not routinely performed in clinical microbiology laboratories, and its misidentification as *K. pneumoniae* is well-documented (21). It should be noted that most of the discordances at the species level are well-known and are limitations described in the instructions for use of both MALDI-TOF databases. Overall, for organisms paired-identified by both instruments, concordance at the genus and species level was remarkably high.

For short incubation blood cultures, significant performance differences between the two systems became evident after 4 h of incubation, especially without formic acid pretreatment. For these samples, the VMP demonstrated a higher identification rate compared to the BS system, both with formic acid (95.0% vs 92.4%, *P* = 0.42) and without (99.2% vs 88.2%, *P* < 0.0001). In summary, the results show that same-day identification of bacterial isolates following a positive blood culture is feasible. Rapid identification of pathogens causing bloodstream infections is clinically crucial, as it allows for the early initiation of targeted infection control measures (3). Identification based on short-term subcultures has been shown to provide accurate clinical results in more than 80% of cases with VITEK MS PRIME and Biotyper system (22). In contrast to the findings above, Grohs et al. reported a lower identification rate for microcolonies grown for 4 h after isolation from blood cultures (23), achieving only 55.5% and 70.2% identification rates by the MBT and VMP systems, respectively. These differences could be explained by the use of formic acid; differences in terms of instrument version, a decreased cut-off for acceptable identification, and the number of h subculture (between 4 hours and 8 hours according to the study). In this study, adding formic acid was shown to significantly increase performance only for Sirius. However, it should be noted that the identification of microcolonies from blood cultures is not recommended by both manufacturers. Bruker proposes a solution for direct identification from positive blood cultures, the MBT Sepsityper Kit (24). Recently, bioMérieux introduced the VITEK MITUBE, a disposable sample preparation device that enables fast identification of Gram-negative microorganisms directly from positive BACT/ALERT blood culture bottles. When considering both the prospective clinical and contrived samples, VITEK MITUBE showed a performance of 95% among claimed species (25).

This study evaluated the workflow characteristics of two MALDI-TOF systems regarding their suitability for routine laboratory procedures. The TTR from process initiation to final identification was significantly shorter for the BS (155 min) compared to the VMP system (191 min). However, the actual HOT when the technician was bound to the process was lower for the VMP (106 min), compared to the BS (155 min). A notable issue with the BS was its recurring difficulty in detecting the BTS validation spot. This frequently required repeating the BTS spot, leading to time consumption and necessitating a technician to be dedicated to the process. Although no issue was observed with the calibrator of the VITEK MS PRIME, it should be noted that in the event of a calibrator acquisition error, all 16 spots of an acquisition group must be re-spotted onto another acquisition group, whereas with the Bruker system, only the BTS needs to be re-spotted. A major difference between the two devices is the VITEK MS PRIME’s new feature, which allows loading and sequential automated measurement of up to 16 slides into the device. This feature explains the shorter HOT (43%) of the VMP and increased walkaway times for the technicians. The advantage of this innovation for laboratory workflow has also been demonstrated in another publication (26). For labs working with only a few slides and a high number of spots prepared before acquisition, the Sirius system may be faster. Thelen et al. have compared Sirius to VITEK MS PRIME with three different scenarios (one slide with 95 spots, one slide with 47 spots, and four slides of 16 spots). They showed that the Sirius system had a shorter turnaround time and hands-on time when a large number of isolates were processed on a single slide, and that the more slides were used, the smaller the difference between the two systems became (9). Here, we aimed to evaluate the total time saved by using the maximum capacity offered by the VITEK MS PRIME system (16 slides); thereby, we showed a lower HOT required for the total process, offering an advantage for large laboratories with high throughput and multiple workbenches.

### Conclusion

Both MALDI-TOF systems evaluated in this study demonstrated excellent agreement in identifying a wide range of different species. Depending on the laboratory organization of the identification process, both systems are well-suited for an efficient workflow and can flexibly be adapted to different workflow settings. However, under the tested conditions, the VITEK MS PRIME demonstrated superior workflow efficiency. Its continuous “load-and-go” operating mode substantially reduced hands-on time and improved throughput, offering a distinct advantage for laboratories processing identifications throughout the day.

## SUPPLEMENTAL MATERIAL

Table S1 spectrum.02981-25-s0001.pdf Species identified by VMP but not by BS.

ASM does not own the copyrights to Supplemental Material that may be linked to, or accessed through, an article. The authors have granted ASM a non-exclusive, world-wide license to publish the Supplemental Material files. Please contact the corresponding author directly for reuse.
