# Supplementary material for: Comparative evaluation of two MALDI-TOF MS systems for microbial identification: accuracy and workflow efficiency in a clinical microbiology laboratory
Source: Microbiol Spectr. 2026 Jan 30;14(3):e02981-25. doi: 10.1128/spectrum.02981-25 (PMC12955377; doi:10.1128/spectrum.02981-25)
Supplement: Table S1 — Species identified by VMP but not by BS. [file spectrum.02981-25-s0001.pdf]

Supplementary data

Supp. Table 1. Species identified by VMP but not by BS

Species	n
Achromobacter sp.	2
Acinetobacter pittii	1
Aerococcus sanguinicola	2
Aeromonas punctata (caviae)	1
Bacillus altitudinis/pumilus	1
Bacillus cereus group	6
Bacillus suptilis	1
Bordetella trematum	1
Brevibacterium luteolum	1
Candida parapsilosis	1
Clostridium innocuum	1
Corynebacterium aurimucosum	4
Corynebacterium glucuronolyticum	1
Corynebacterium striatum	2
Corynebacterium tuberculostearicum	1
Corynebacterium urealyticum	1
Enterobacter soli	2
Facklamia laguida	1
Geotrichum cadidum/klebahnii	1
Helicobacter pylori	2
Kerstersia gyiorum	1
Klebsiella oxytoca	1
Klebsiella pneumoniae	3
Myroides odoratus	1
Pantoea ananatis	1
Peptostreptococcus anaerobius	1
Proteus mirabilis	1
Proteus penneri	1
Providencia stuartii	2
Pseudomonas aeruginosa	2
Roseomonas mucosa	1
Rothia mucilaginosa	1
Saccharomyces cerevisiae	1
Staphylococcus cohnii ssp cohnii	2
Staphylococcus cohnii ssp urealyticus	1
Staphylococcus haemolyticus	1
Staphylococcus lugdunensis	1
Staphylococcus sciuri	1
Staphylococcus simulans	1
Stenotrophomonas maltophilia	1
Streptococcus anginosus	2
Streptococcus pneumoniae	1

Streptococcus pyogenes	2
Streptococcus salivarius/vestibularis	1
Streptococcus sanguinis	1
Streptococcus sp.	1
Winkia neuii	1
